# What has been the impact of western governments’ laws and policies on the mental health of asylum seekers and refugees? A systematic-narrative hybrid literature review

**DOI:** 10.1016/j.jmh.2025.100382

**Published:** 2025-12-05

**Authors:** Imen El Amouri, Tihomir Sabchev

**Affiliations:** Tilburg University, the Netherlands

**Keywords:** Asylum seekers, Refugees, Law, Policy, Mental health

## Abstract

This article reviews research on the causal impact of Western governments’ laws and policies on the mental health of asylum seekers and refugees. The systematic-narrative hybrid literature review yielded 34 studies using quantitative, qualitative and mixed methods approaches, focusing almost exclusively on restrictive and deterrence-oriented measures. The synthesis shows that, over the last two decades, Western governments’ laws and policies around detention, access to basic rights, asylum procedure, and reception have had a substantial and almost exclusively negative impact on the mental health of protection seekers. Based on our findings, we urge legislators and policymakers to consider the long-term consequences and costs of the laws and policies they introduce, within and beyond the realm of mental health. In addition, we highlight the need for more research on governmental measures that are likely to have a positive impact on the mental health of asylum seekers and refugees.

## Highlights

• Western laws and policies negatively affect asylum seekers' and refugees' mental health.
• Research focuses on deterrence policies, neglecting positive mental health measures.
• Uncertainty, isolation, and dehumanization are key elements behind mental health harm.
• Access to healthcare and community support has positive impact on mental health.
• Policymakers must weigh long-term mental health costs of deterrence measures .

## 1 Introduction

Since the end of the Cold War, Western governments have engaged in an incessant series of legal and policy reforms in the area of asylum and refugee reception (FitzGerald, 2019; Gibney and Hansen, 2005). Regardless of their objectives, such reforms often impact the mental health of asylum seekers and refugees (ASRs). Numerous studies have shown that protection seekers arrive with high rates of psychological problems due to traumatic experiences (Bogic et al., 2015; Haasen et al., 2008), and must cope with post-arrival stressors, including discrimination, family separation, and language barriers (Miller et al., 2018; Schiess-Jokanovic et al., 2021). Legal and policy changes around detention upon arrival (Cleveland et al., 2018; Cleveland and Rousseau, 2013; Coffey et al., 2010), confinement in reception facilities with poor living conditions (Bjertrup et al., 2018; Eleftherakos et al., 2018), or temporary status with limited access to rights (Fleay and Hartley, 2016; Johnston et al., 2009) can further harm ASRs mental health. In contrast, measures that facilitate ASRs’ access to rights and services, such as early and non-bureaucratic access to healthcare, can have positive effects on newcomers’ mental health (Jaschke and Kosyakova, 2021). In the long run, through their impact on ASR’s mental health, laws and policies can either hinder or facilitate newcomers’ settlement (Walther et al., 2021).

Previous attempts to systematize and/or synthesize findings on the impact of laws and policies on ASR’s mental health have several shortcomings. First, earlier literature reviews focus on specific legal or policy areas (e.g., immigration detention or asylum procedure (Hornfeck et al., 2022; Mares, 2021; Robjant et al., 2009; Ryan et al., 2009; von Werthern et al., 2018)), specific groups of immigrants (e.g., undocumented immigrants (Martinez et al., 2015)), specific mental health interventions (Murray et al., 2010), or broader post-migration stressors (Gleeson et al., 2020; Hajak et al., 2021). Second, such reviews often include either quantitative or qualitative studies, synthesizing findings from one approach without integrating the other (Hornfeck et al., 2022; Juárez et al., 2019; von Werthern et al., 2018). Third, earlier reviews do not distinguish between studies that evidence the *causal* impact of laws and policies on ASRs’ mental health and studies that merely evidence the *association* between the two. Consequently, the link between legal and policy reforms and newcomers’ mental health remains unclear.

The aim of this study was to address these shortcomings. To this end, we present findings from a systematic-narrative hybrid literature review that focuses on answering the question *what has been the impact of Western governments’ laws and policies on the mental health of asylum seekers and refugees?* Our strategy is guided by a broad understanding of law, policy, and mental health, as well as a broad conceptualization of ASRs as individuals who have applied for international protection, regardless of the outcome (therefore including asylum seekers, refugees, beneficiaries of other types of protection, rejected asylum seekers). Furthermore, our review includes only studies that make a compelling argument for the *causal* impact of laws and policies across different domains (e.g., asylum, reception, welfare, etc.) on ASRs’ mental health. By “compelling argument” we refer here to implicit or explicit causal claims that are based on rigorous methodology and go beyond merely describing associations. The adoption of this broad and simultaneously narrow perspective in the search strategy enables us to uncover and synthesize the effect of Western governments’ laws and policies on newcomers’ mental health, and also helps us find some conceptual and empirical gaps in the literature.

Before moving on, it is imperative to immediately address any concerns regarding our exclusive interest in the *causal* impact of laws and policies on ASRs’ mental health. Evidencing causality in this context is undoubtedly a notoriously difficult, and yet not an impossible task (Fontaine and Geva-May 2022; Kay and Baker, 2015). We acknowledge that researchers from different disciplinary perspectives subscribe to different research paradigms, and consequently may have very different opinions on how causation ought to be inferred (Baškarada and Koronios, 2018; Tacq, 2011). Although engaging in this debate falls beyond the intention and scope of this article, we acknowledge that both positivist and interpretivist approaches can be used for causal inference depending on one’s research design (research setting, number of observations, mechanisms linking cause and effect, etc.). Therefore, we have included in our review studies that in our assessment evidence the causal impact of laws and policies on ASRs’ mental health, regardless if these studies rest on positivist or interpretivist foundations, or at times even on a combination of the two. At the same time, we have excluded from our review studies in which the author(s) explicitly state that causal claims cannot be made based on their findings.

Our search strategy and quality assessment shortlisted 34 studies examining the impact of Western governments’ laws and policies on ASRs’ mental health across four domains: detention, access to basic rights, asylum procedure, and reception. Furthermore, some of the studies examined the cumulative effect of broader legal and policy frameworks that included elements from two or more of the above domains. Our narrative synthesis shows that most research to date has focused on deterrence-oriented measures, highlighting how such measures can exacerbate uncertainty, social isolation and dehumanization, hence harming ASRs’ mental health. In contrast, few studies shed light on the positive impact of policies facilitating early psychological support for ASRs. We conclude with suggestions for future research and recommendations for policymakers.

## 2 Methodology

To collect, analyze, and synthesize evidence on the causal impact of Western governments’ laws and policies on ASRs’ mental health, we use a systematic-narrative hybrid approach (Turnbull et al., 2023). Our literature review combines methodological features typical for a systematic review, such as detailed search protocol and inclusion/exclusion criteria, with an interpretative synthesis of data from the extracted studies (Greenhalgh et al., 2018). Given our broad focus on qualitative and quantitative studies and our specific research question, this hybrid approach enhances methodological robustness and allows for flexibility in presenting and discussing our findings (Turnbull et al., 2023).

### 2.1 Search strategy

Initially, the first author identified relevant databases following standard literature review procedures. This resulted in the selection of six databases: Web of Science, Scopus, PsycINFO, PubMed, NexisUni, and Legal Intelligence. Subsequently, twenty-seven search terms were combined in a search matrix using Boolean operators (see Appendix A). Search strategies were adapted for each database. For the legal databases (NexisUni and Legal Intelligence), simplified queries were necessary due to limited Boolean and truncation functions. This was supplemented by hand searching the reference lists of identified studies conducted by both authors. The identification and screening followed the PRISMA guidelines, a widely recognized method for conducting systematic reviews (Page et al., 2021).

### 2.2 Inclusion and exclusion criteria

To be included in the review, each study had to: i) be peer-reviewed qualitative, quantitative, or mixed-method; ii) be published between 1992–2023;^1^ iii) include ASRs of any gender over 18 years old residing in Western countries;^2^ iv) be in English; v) report on the mental health of ASRs; and vi) provide evidence for the causal impact of specific laws/policies on ASRs’ mental health. We excluded from the review: i) studies that focus on refugee children and unaccompanied minors, as the respective legal and policy frameworks differ substantially and would draw us beyond the scope of our review; ii) studies that investigate specific physiological diseases (e.g., HIV, diabetes, coronary heart diseases) iii) letters, commentaries, replies, conference abstracts, book chapters, literature reviews, and publications that do not feature a title or an abstract.

### 2.3 Study selection

**Fig. 1:**
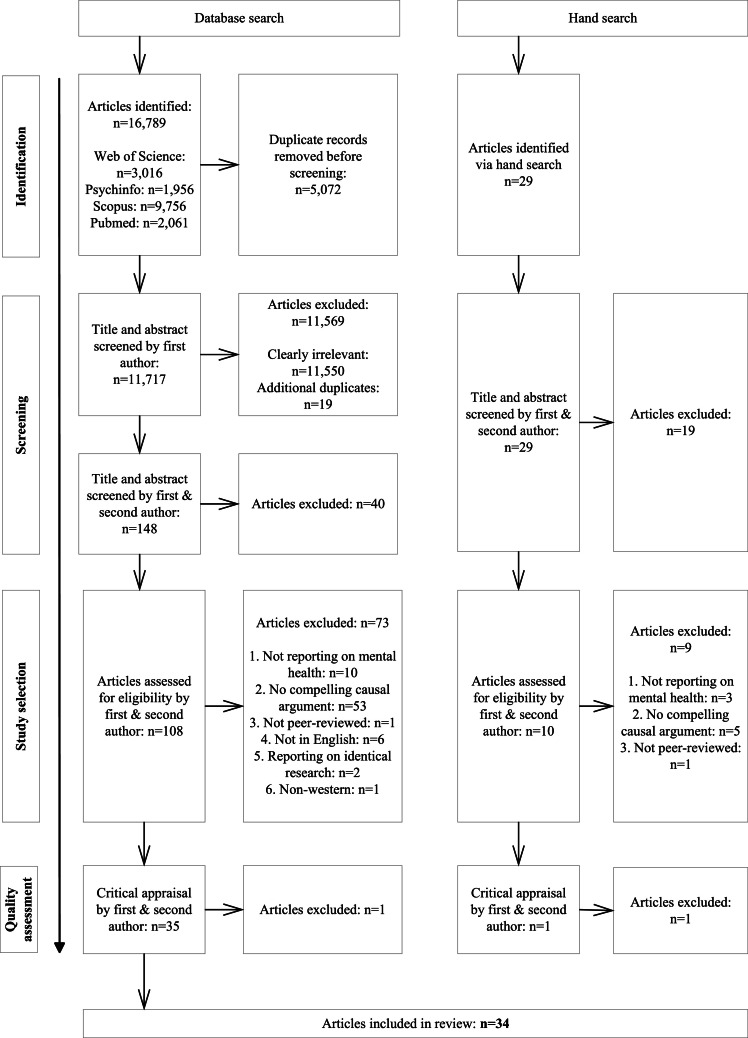
PRISMA flow diagram summarizing the identification, screening, study selection, and quality assessment process.

We used a three-step study screening and selection process (see Fig. 1). First, the first author conducted a preliminary title and abstract screening of studies from selected databases (*n* = 11,717) and removed the studies that were deemed irrelevant based on the inclusion and exclusion criteria. This narrowed the sample to 148 studies, which were uploaded in Covidence for streamlined screening and data extraction. Second, the titles and abstracts of these 148 studies were screened independently by the first and the second author, resulting in the further exclusion of 40 studies. Third, the remaining 108 studies were retrieved, read in full by both authors, and assessed for eligibility according to the exclusion and inclusion criteria, resulting in 35 articles. To reduce subjectivity in reporting reasons, we applied a prioritization and sequential exclusion approach (see details under the “Study selection” step on Fig. 1). Discrepancies during screening were resolved by consensus. An additional 29 studies identified through hand-searching were screened, resulting in 10 more studies, only one of which met our criteria.

The exclusion of studies on the basis of not providing evidence for the *causal* impact of specific laws/policies on ASRs’ mental health merits further clarification. According to our assessment, 58 studies did not present a compelling argument for the presence of such causal relationships. Appendix B provides detailed information on the reasons for excluding each one of these studies from our review. In a nutshell, in some of the excluded studies, the authors explicitly state that their claims are not causal, even if their study evidences an association between laws/policies and ASRs’ mental health. In other studies, the authors acknowledge different limitations in their research design and do not present their results in causal terms. In yet other studies, the relationship that the authors evidence is not between ASRs’ mental health and specific laws/policies, but between mental health and other variables/factors (e.g., employment status, family reunification, etc.).

### 2.4 Quality assessment

The quality assessment of selected studies from the database (*n* = 35) and hand search (*n* = 1) was conducted independently by each author and discussed in case of discrepancies. In conducting the critical appraisal, we used the 16-item quality assessment tool for reviewing studies with diverse design (QATSDD) (Sirriyeh et al., 2012). The tool includes 14 criteria for qualitative and quantitative studies, with all 16 criteria applying to mixed methods studies. Each criterion (e.g. explicit theoretical framework, clear description of research setting, etc.) is assessed on a four-point scale, with a score of 3=complete, 2=moderately, 1=very slightly, or 0=not at all. This gives an overall maximum score of 48 for mixed methods or 42 for qualitative/quantitative studies. We applied a quality threshold of 50 % of the maximum score (as a subjective but pragmatic adaptation of the assessment tool to ensure minimum quality) and a 20 % margin of divergence between the assessment of first and the second author (with further discussion in cases of larger discrepancy until reaching an agreement between the two authors). Only 2 studies were excluded for not meeting the 50 % threshold. This resulted in shortlisting 34 studies for our review.

### 2.5 Data extraction, analysis and synthesis

We extracted data from each study, including the country of study, methodology, study setting, number of participants and their characteristics, law/policy, outcome measures, and finding of interest (see Table 1). The first author used Atlas.ti to analyze the 34 shortlisted studies. Segments of text were coded inductively and deductively around three broad themes: (a) conceptualization/operationalization of mental health, (b) legal/policy domain, and (c) causal mechanism linking law/policy and mental health impact. Codes were subsequently grouped into broader families which provided the analytical categories for cross-study synthesis.

**Table 1:** Data extracted from the reviewed studies.

*Author & Year of publication*	*Country of study*	*Methodology*	*Study setting*	*Participants' characteristics*	*Law/policy*	*Outcome measures*	*Finding of interest*
van Eggermont Arwidson et al. 2022	Sweden	Qualitative	Accommodation centers for asylum seekers	*N* = 14; Diverse group of asylum seekers (age, educational level, gender, marital status, time in Sweden, country of origin)	Reception in state-provided accommodation centers	Qualitative interviews	The asylum procedure along with the stressors of living in accommodation center pave a pathway to poor mental health, with risks of long-lasting mental health consequences for asylum seekers.
Bjertrup et al. 2018	Greece	Mixed method	Four refugee camps on mainland Greece, a squatted building in Athens, and the reception and identification center on Samos	*N* = 1293 (1293 in the quantitative component, and 83 of them in the qualitative component); Diverse group with different migration statuses residing in the four camps, the squatted building, or the identification center	Asylum and reception policy (broadly understood)	Refugee Health Screener-15 Qualitative interviews	The Greek reception and asylum policy causes psychological distress and social suffering to refugees (acknowledging that this distress/social suffering is also inseparable from the broader social, political, and institutional context).
Caroppo et al. 2021	Italy	Quantitative	Research center at a hospital	*N* = 180; Diverse group of (mostly single) refugees and asylum seekers	Transfers to the first country of entry under the Dublin III Regulation	DSM-IV-TR SCID-I Axis Clinical Global Impression Scale List of Migration Experiences (LiMEs)	Asylum seekers returned to Italy under the Dublin Regulation may develop "Dublin Migration Syndrome", characterized by significant impulsivity and aggressive behaviors within a twilight state of consciousness and fragmentary aspects of identity, up to shape a psychotic picture.
Chaffelson et al. 2023	United Kingdom	Qualitative	Asylum interviews with the Home Office	*N* = 8; English-speaking refugees who have successfully gone through the asylum procedure in the UK and have been granted permission to stay	Asylum procedure and asylum interviews	Qualitative interviews	The asylum procedure in the UK and the Home Office interviews can have negative psychological impacts on refugees, which can endure even after “leave to remain” has been granted.
Cleveland et al. 2018	Canada	Qualitative	Detention centers in Montreal and Toronto	*N* = 81; Detained asylum seekers	Detention of asylum seekers	Qualitative interviews	Detention, even for brief periods in relatively adequate conditions, is detrimental to asylum seekers’ mental health, largely due to the combined effect of symbolic violence and disempowerment.
Cleveland and Rousseau 2013	Canada	Quantitative	Detention centers in Montreal and Toronto, as well as community setting (for the non-detained asylum seekers)	*N* = 188; Detained (*n* = 122) and non-detained asylum seekers (*n* = 66) in Montreal and Toronto, whose refugee claims have not been adjudicated	Detention of asylum seekers	HTQ HSCL-25	For asylum seekers, even brief detention is associated with increased psychiatric symptoms.
Coffey et al. 2010	Australia	Mixed method	Detention centers	*N* = 17; Refugees who have been held in immigration detention	Detention of asylum seekers	Qualitative interviews HTQ HSCL-25 World Health Organization Quality of Life-BREF	Prolonged detention causes long-term psychological harm to asylum seekers.
Domínguez et al. 2022	United States	Qualitative	Community-based	*N* = 7; Asylum seekers from Honduras and Venezuela	Apprehension and detention of asylum seekers	Qualitative interviews	Asylum seekers suffer victimization during their apprehension and detention, which has adverse effects on their mental health.
Eleftherakos et al. 2018	Greece	Qualitative	Reception center	*N* = 76; Afghan, Syrian, and Congolese migrants residing in Kara Tepe and Moria camp on Lesvos (*n* = 67) and Médecins Sans Frontières’ health providers operating inside Kara Tepe and Moria camp (*n* = 9)	EU-Turkey deal, asylum seekers' geographical restriction of movement and reception on the island of Lesvos	Qualitative interviews	The EU-Turkey deal facilitates an institutional abuse on Lesvos and creates an environment where traumatic stressors are continuous, while the provision of adapted mental health services for the migrant population on the island is inadequate/insufficient.
Fleay and Hartley 2016	Australia	Qualitative	Community-based	*N* = 29; Diverse group of asylum seeker who arrived to Australia by boat	Bridging Visa E policy: denying the right to work and providing limited (financial) support to asylum seekers released from immigration detention into community-based arrangements	Qualitative interviews	The Australian Bridging Visa E policy has a negative mental health impact on at least some asylum seekers.
Hocking et al. 2015	Australia	Quantitative	Community-based	*N* = 131; Community-based asylum seekers (*n* = 98) and refugees (*n* = 33)	Refugee determination process & the changing health and employment statuses associated with it	HTQ-Revised, HSCL-25 Psychiatric Epidemiology Research Interview Demoralization Scale (PERI-D)	Living in the community with work rights and access to health cover significantly improves psychiatric symptoms in forced migrants irrespective of their protection status.
Hollis 2019	United Kingdom	Qualitative	Community-based	*N* = 9; Formerly detained immigrants, most of them asylum seekers	Detention of asylum seekers	Qualitative interviews	Asylum seekers may experience incredulity and cognitive dissonance at being detained, which may lead them to feel powerless, doubt themselves and their worldviews, and ruminate about their uncertain futures.
Jaschke and Kosyakova 2021	Germany	Quantitative	Regional healthcare system	*N* = 5922; Refugees who arrived in Germany between 2013 and 2016	Electronic Health Card for refugees	PHQ-4 (Patient Health Questionnaire-4), Mental component summary scale of IAB-BAMF-SOEP Survey of Refugees	The provision of early, easy, and nonbureaucratic access to healthcare services, as implemented by the Electronic Health Card, has considerable positive effects on refugees’ mental health.
Johnston et al. 2009	Australia	Mixed method	Community-based	*N* = 131; Iraqi Temporary Protection Visa refugees (*n* = 71) and Iraqi Permanent Humanitarian Visa refugees (*n* = 60) residing in Melbourne	Temporary Protection Visa policy and Permanent Humanitarian Visa policy	The Medical Outcomes Study Social Support Scale (MOS-SS), The Perceived Constraints subscale of the Lachman and Weaver Sense of Control Scale, The State-Trait Anger Expression Inventory (STAXI)	The Australian Temporary Protection Visa policy has negative impact on the psychological health of refugees.
Kahn and Alessi 2017	Canada	Qualitative	Asylum interview	*N* = 29; LGBT forced migrants (*n* = 7) and service providers (*n* = 22), including attorneys, mental health providers and advocates	Asylum interview	Qualitative interviews	Early disclosure of sexual violence, compressed timelines for filing a refugee claim, and coming out before they are ready may contribute to negative psychological outcomes and identity confusion for LGBT refugee claimants.
Laban et al. 2004	the Netherlands	Quantitative	Community-based and state-provided accommodation	*N* = 294; Iraqi asylum seekers who have resided for less than 6 months in the Netherlands (*n* = 143) and Iraqi asylum seekers who have been living in the Netherlands for at least 2 years (*n* = 151)	Long asylum procedure	World Health Organization Composite International Diagnostic Interview (CIDI), version 2.1	Prolonged asylum procedures lead to much higher levels of psychiatric disorders such as depression and anxiety in asylum seekers.
Llewellyn 2021	United States	Qualitative	Community-based	*N* = 18; LGBTQ asylum seekers from Caribbean and African countries	Policies aimed at reducing fraudulent asylum claims (delayed work authorization and shifting timelines)	Qualitative interviews	Work restrictions and shifting asylum procedures and timelines may have adverse effects on the mental health of LGBTQ asylum seekers.
Mansouri and Cauchi 2007	Australia	Qualitative	Community-based	*N* = 10; Temporary Protection Visa holders from Iraq, Afghanistan and Syria living in and around Melbourne	Detention and Temporary Protection Visa policy	Qualitative interviews	Australia's detention and Temporary Protection Visa policy is perpetuating and exacerbating refugees’ mental illness.
Mercado et al. 2022	United States	Mixed method	Humanitarian respite center	*N* = 6; South American asylum-seeking families	Migrant Protection Protocols (MPP)	Brief Symptom Inventory (BSI-18) HTQ-R	The MPP policies subject migrating families seeking asylum in the US to continuous trauma exposure and harsh living conditions leading to psychological distress symptoms.
Minero et al. 2022	United States	Qualitative	Community-based	*N* = 30; LGBTQ refugees from South America	Detention of asylum seekers	Qualitative interviews	By engaging in dehumanization, abuse, and transphobic practices, the U.S. detention system inflicts trauma, anxiety, depression, and suicidal thoughts on LGBTQ asylum seekers, leading some to prefer self-deportation.
Nickerson et al. 2011	Australia	Quantitative	Community-based	*N* = 97; Mandean refugees	Temporary Protection Visa policy	HSCL-25 HTQ symptom subscale PMLDC MHR-QOL	Restriction of rights and access to services under the Temporary Protection Visa Policy negatively affect the mental health of refugees.
Pérez-Sales et al. 2022	Greece	Quantitative	Moria Reception Center on Lesvos	*N* = 160; Diverse sample of refugees (age, gender, ethnic background) residing in the Moria refugee camp	Reception policy	Torturing Environments Scale	From a medical-psychological perspective, the Moria refugee camp constitutes a torturing environment that causes suffering and mental health deterioration.
Sagbakken et al. 2022	Norway	Qualitative	Asylum reception center	*N* = 21; Diverse group of rejected asylum seekers	Policies aimed at reducing access to rights for rejected asylum seekers	Qualitative interviews	The gradual loss of rights, opportunities and finances of rejected asylum seekers is experienced as a form of violence that leads to extreme mental and social suffering.
Schock et al. 2015	Germany	Quantitative	Treatment Center for Torture Victims	*N* = 47; Asylum seekers with PTSD	Asylum interview	DSM-IV PTSD HSCL-25	The asylum interview leads to significant increase in posttraumatic intrusions and a decrease in posttraumatic avoidance and hyperarousal symptoms.
Scott 2018	Germany	Qualitative	Shelter for rejected asylum seekers	*N* = 10; Black African rejected asylum seekers	“Duldung” (Tolerated Stay Permit) and the associated welfare provisions under the Asylum Seekers Benefit Act	Qualitative interviews and participant observation	Rejected asylum seekers living on Duldung experience a range of stressors arising out of social arrangements structured by the applicable laws and policies, which leads to psychological distress and mental health problems.
Sobhanian et al. 2006	Australia	Quantitative	Community-based	*N* = 150; Former refugee detainees from the Woomera Detention center	Detention of asylum seekers	Truncated Assessment of Self-Destructive Thoughts (TASDT), The Quality of Life Inventory, The Suicidal Ideation Scale, The Profile of Mood States	Former refugee detainees from the Woomera Detention Centre manifest significantly improved psychological functioning when living in the Australian community as compared to living in the detention center.
Steel 2004	Australia	Quantitative	Detention facility	*N* = 34; Ten families (14 adults and 20 children) from one ethnic group in an Australian detention center.	Detention of asylum seekers	Detention experiences checklist, Detention symptom checklist, Schedule for Affective Disorders and Schizophrenia for School-Age Children – Present and Lifetime Version (K-SADSPL). Structured Clinical Interview for DSM-IV Axis I Disorders. Parenting questionnaire	Detention appears to be injurious to the mental health of asylum seekers.
Steel et al. 2011	Australia	Quantitative	Service of the Treatment and Rehabilitation of Torture and Trauma Survivors (STARTTS)	*N* = 104; Afghan and Irani refugees granted Permanent Protection Visas (PPV) prior to entering Australia (*n* = 57) or released from immigration detention on a Temporary Protection Visa (TPV) (*n* = 47)	Temporary Protection Visa & Permanent Protection Visa policy	HTQ HSCL-25 The general health questionnaire The Penn State Worry questionnaire, PMLDC	Refugees on TPV show a pattern of growing mental distress, ongoing resettlement difficulties, social isolation, and difficulty in the acculturation process compared to refugees on PPV.
Steel et al. 2006	Australia	Quantitative	Community-based	*N* = 24; Arabic speaking Mandean refugees in Sydney	Detention and Temporary Protection Visa policy	HTQ HSCL-25, MCS SF-12, PMLDC	TPV and prolonged detention policies contribute substantially to ongoing depression, PTSD, and mental health-related disability in refugees.
Targarona Rifa and Donà 2021	United Kingdom	Qualitative	Community-based	*N* = 52; Asylum seekers and refugees (*n* = 47), key informants from asylum organizations (*n* = 5)	Restriction of access to employment for asylum seekers	Qualitative interviews	The hostile environment and the prohibition to work in the UK has a negative emotional impact on asylum seekers.
Walsh et al. 2022	Australia	Qualitative	Community-based	*N* = 15; Refugees and asylum seekers on a Temporary Protection Visa	Temporary Protection Visa and Safe Haven Enterprise Visa (SHEV)	Qualitative interviews	Precarious visa status directly and negatively impacted ASRs’ mental health, prompted in particular by difficulties finding employment, difficulties within employment, lack of government supports, precarious finances, and ongoing immigration insecurity.
Watters et al. 2022	Ireland	Qualitative	Community-based	*N* = 9; Resettled refugees	Irish Refugee and Protection Programme (IRPP)	Qualitative interviews	Refugees resettled through the Irish Refugee and Protection Program and living in temporary accommodation experienced feeling lack of control over their circumstances, a sedentary lifestyle, and a lack of meaningful activities, ultimately leading to boredom, frustration and anxiety.
Whitehouse et al. 2021	Belgium	Qualitative	Reception centers	*N* = 41; Asylum seekers residing in reception centers (*n* = 29), MSF project staff (*n* = 4), Fedasil staff (*n* = 8)	Reception policy	Qualitative interviews	Daily stressors linked to the reception conditions such as the absence of privacy, overcrowding, lack of meaningful activities and psychological support combined with the protracted uncertainty of the asylum procedure lead to stress, isolation, sense of abandonment and feelings of worthlessness among asylum seekers.
Willmann-Robleda 2022	Norway	Qualitative	Reception centers	*N* = 9; Asylum seeking women	Asylum procedure	Qualitative interviews	Different elements of the asylum procedure in Norway (seemingly endless wait and uncertainty, limited ability to control circumstances, the limitations to engage in meaningful activities, financial limitations and forced spatial (im)mobility) negatively impact the psychological well-being of asylum seeker women.

## 3 Results

This section presents the results of our hybrid systematic-narrative literature review. Before discussing the impact of laws and policies on ASR’s mental health, we summarize the study descriptives and mental health conceptualization in the reviewed studies.

### 3.1 Study descriptives

**Fig. 2:**
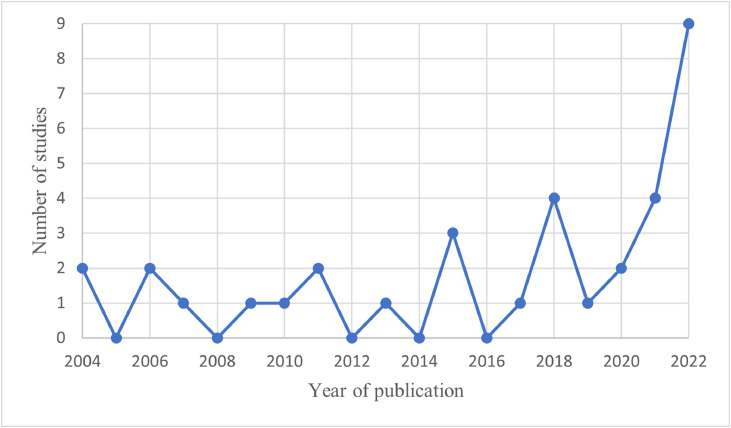
Number of reviewed studies per year of publication^3^.

The 34 shortlisted studies were published between 2004 and 2022. As illustrated in Fig. 2, scholarly evidence on the causal impact of laws and policies on ASR’s mental health has been limited but relatively stable over the examined period and has peaked in 2022.

The reviewed studies used qualitative (*n* = 18), quantitative (*n* = 12), and mixed-methods approaches (*n* = 4). Most studies were conducted in Australia (*n* = 11), followed by the US (*n* = 4), the UK (*n* = 3), Canada (*n* = 3), Germany (*n* = 3), Greece (*n* = 3), Norway (*n* = 2), Ireland, Italy, Belgium, the Netherlands, and Sweden (*n* = 1). Research settings included community-based settings, reception centers, temporary accommodation facilities, detention centers, health-related facilities (see Table 1).

The studies reported data on 9256 participants. Most studies (*n* = 22) featured heterogenous samples in terms of age, gender, and country of origin of the participants. In contrast, twelve studies focused on more homogenous groups, including participants with specific ethnicity or nationality (*n* = 4), LGBTQ asylum seekers (*n* = 3), male (*n* = 1) or female ASRs (*n* = 1), asylum seeking families (*n* = 2), and asylum seekers with PTSD (*n* = 1). Three studies compared different ASR groups: formerly detained with resettled refugees (Steel et al., 2011), detained with non-detained (Cleveland and Rousseau, 2013), and permanent with temporary visa holders (Johnston et al., 2009).

### 3.2 Conceptualization and operationalization of mental health

The analysis of the studies revealed a shortcoming in conceptualizing mental health. Most reviewed studies did not define mental health, with only eight studies providing explicit statements on how the authors conceptualize mental health. This is an omission that poses some limitations to our findings, but more importantly also raises validity questions in relation to a large part of the available research on the causal impact of laws and policies on ASRs’ mental health.

The studies used diverse instruments to assess mental health impacts (see “Outcome measures” in Table 1). We observed a general (although rather broad) consensus around what constitutes an indication of mental health impact. On the one hand, quantitative studies – including the quantitative component of mixed-method studies – predominately employed a psychiatric and psychosocial lens, acknowledging the interplay between biological, psychological, social, and environmental factors. In addition to the primary tools for assessing mental health impacts in these studies, which were the Harvard Trauma Questionnaire (*n* = 7) and the Hopkins Symptom Checklist (*n* = 7), we identified multiple other less common measuring tools, such as the Suicidal Ideation Scale, the Penn State Worry Questionnaire, and the World Health Organization Quality of Life-BREF (WHOQOL-BREF). On the other hand, qualitative studies – including the qualitative component of mixed-method studies – relied on ASRs’ and in some cases also on practitioners’ narratives to uncover mental health impacts such as stress, intense worry, anxiety, panic attacks, flashbacks, intrusive thoughts, nightmares, depression, hopelessness, self-harm, and suicidal thoughts. Our analysis shows that there is a significant overlap between qualitative and quantitative indicators of mental health, suggesting a broad common understanding of mental health impacts across studies.

### 3.3 Impact of laws and policies on ASR’s mental health

**Fig. 3:**
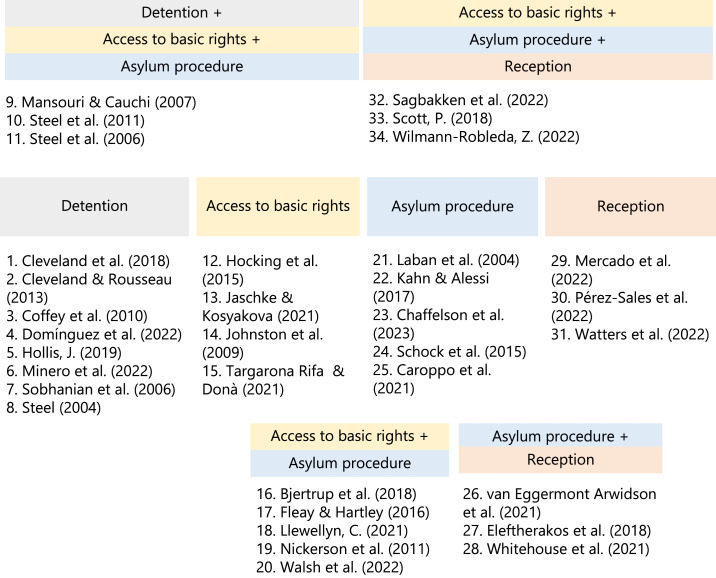
Allocation of reviewed studies across legal and policy domains and additional clusters.

Our review of the 34 shortlisted studies revealed that ASR’s mental health is affected by various laws and policies introduced by Western governments. Causal claims were explicitly made by the authors of 3 studies (Jaschke and Kosyakova, 2021 based on quasi-experimental research design; Minero et al., 2022 based on participant accounts; Pérez-Sales et al., 2022 based on quantitative evidence that Moria refugee camp constitutes a torturing environment) and only inferred in the remaining 31 studies. We identified four overarching legal and policy domains – detention, access to basic rights, asylum procedure, and reception – with 20 studies fitting these categories. The remaining 14 studies examined the cumulative impact of broader legal/policy frameworks that included elements from multiple domains (e.g., asylum procedure *in conjunction with* reception). These studies are presented in four additional clusters (see Fig. 3). The following sections present the findings across each of the four main domains and the four additional clusters.

#### 3.3.1 Detention

Eight studies address the mental health implications of ASRs’ detention in Western jurisdictions, focusing particularly on Australia’s detention regime. The studies vary in settings, populations, and methods: two involve only detained participants, five only formerly detained participants, and one compares detained and non-detained asylum seekers.

Demographically, the studies mostly report on diverse groups of ASRs (*n* = 5), but also families (*n* = 1), LGBTQ refugees (*n* = 1), and asylum seekers from Honduras and Venezuela detained in the US (*n* = 1). They examine both short-term and long-term detention effects, ranging from 24 h to over three years. Mental health impacts are assessed through self-reporting using clinically validated rating scales (*n* = 3), qualitative interviews (*n* = 4), or both (*n* = 1). Five studies report symptoms of anxiety and depression, with two also noting PTSD symptoms.

All eight studies show unequivocally the universal injurious effect of detention on ASRs’ mental health. In terms of causal mechanisms, detention environments are commonly characterized by prison-like austerity, social isolation, and rudimentary healthcare provision, which engender posttraumatic stress, depression, and anxiety (Cleveland and Rousseau, 2013), poor self-worth and social withdrawal (Coffey et al., 2010), and cognitive dissonance in detained individuals (Hollis, 2019). One study links physical and psychological violence in detention to long-term mental health traumas (Dominguez et al., 2022). Another study on families who have spent protracted periods in detention draws a similar picture and argues that exposure to traumatic experiences and different forms of victimization in detention increases significantly psychiatric disorders in parents and children alike (Steel et al., 2004). Importantly, even brief detention in relatively adequate conditions appears to have negative impact on ASRs’ mental health, largely due to the combined effect of symbolic violence and disempowerment (Cleveland et al., 2018). Lastly, three studies report on suicidal ideation (Hollis, 2019; Minero et al., 2022; Sobhanian et al., 2006), with one of them showing improvement in the psychological status of asylum seekers after release from detention (Sobhanian et al., 2006).

#### 3.3.2 Detention, access to basic rights, and asylum procedure

Three of the reviewed studies (Mansouri and Cauchi, 2007; Steel et al., 2011, 2006) examine the combined impact of detention, limited access to basic rights, and asylum procedures on ASRs’ mental health. These studies focus on the Australian Temporary Protection Visa (TPV) policy and show harmful impact for ASRs’ mental health. Under the TPV policy, formerly detained refugees are given a temporary residence permit (with asylum applications re-examined after 3 years of residence), with reduced socio-economic rights and no right to family reunification (Mansouri and Cauchi, 2007).

The studies in this cluster consistently identify the uncertainty of ASRs’ temporary legal status in Australia as a primary driver of adverse mental health outcomes. For example, Mansouri and Cauchi (2007) found that detention, limited work access, and shifting timelines under the TPV policy exacerbated pre-arrival trauma and contributed to chronic stress, leading to or worsening PTSD, anxiety, and depression. Steel et al. (2006) highlighted that prolonged detention followed by temporary protection increases the risk of ongoing depression, PTSD, and mental health-related disability, even after controlling for other risk factors such as gender, age, extent of past traumas, length of residency, and family separation.

One of the studies in this cluster used a longitudinal design to compare the mental health outcomes of formerly detained refugees holding temporary visas with resettled refugees holding permanent visas (Steel et al., 2011). The study found that refugees subjected to the Australian temporary visa policy are likely to experience persisting or worsening psychiatric symptoms over time due to the instability and insecurity associated with their precarious legal status, the need to re-examine their asylum claims after 3 years, their detention experiences, and their poor access to healthcare, work, language classes, and housing. In contrast, refugees granted permanent residence visas who have a more stable and secure status tend to show improvements in their mental health over time (Steel et al., 2011).

#### 3.3.3 Access to basic rights

We identified four studies focused on laws and policies affecting ASRs’ access to basic rights, such as access to employment, healthcare, and education. It is within this domain that we found the only examples of studies showing a positive impact of legal and policy measures on ASRs’ mental health.

The study of Jaschke and Kosyakova (2021) provides a compelling argument of how a policy facilitating access to healthcare can positively impact ASRs mental health. Using a quasi-experimental design, the authors studied the introduction of electronic health cards (eHCs) that provided ASRs immediate, non-bureaucratic access to healthcare in Germany. The findings – explicitly framed in causal terms – show significant improvements in ASRs’ psychological well-being due to easier access to mental health services, which mitigates post-migration stress. The research of Hocking et al. (2015) corroborates these findings noting that health coverage reduces post-traumatic stress and demoralization, though not depression symptoms.

Access to work is another key theme. Hocking et al. (2015) also found that granting work rights contributes to a recuperative social environment and leads to positive changes in ASR’s psychiatric morbidity, reducing PTSD symptoms in particular. At the same time, however, the authors also note that work rights alone do not reduce depression symptoms. Similarly, Targarona Rifa and Donà (2021) reported that prohibiting work in the UK causes burdens like embarrassment, frustration, sadness, and anger, which in some cases lead to distress and depression. Johnston et al. (2009) concluded that denying core economic and social rights (access to work, education, and language classes) under the Australian TPV policy adversely affects refugees’ mental health.

#### 3.3.4 Access to basic rights and asylum procedure

Five studies examined the mental health impact of laws regulating access to basic rights *in conjunction with* asylum procedures, all detecting negative effects on ASRs’ mental health.

Fleay and Hartley (2016) found that prohibiting work for asylum seekers in Australia and the consequential reliance on minimal financial support leads to prolonged periods of inactivity. This is compounded by ongoing uncertainty around asylum seekers’ protection claims, ultimately resulting in exacerbated feelings of anxiety, sadness, and fear. These findings are in line with the results of Nickerson et al. (2011), which show that the transition from temporary protection visa (i.e., limited access to basic rights and uncertainty regarding the outcome of the asylum claim) to permanent residency status (i.e., broad access to rights and services and secure status) markedly improves the mental health of Mandean refugees in Australia. Similarly, the study of Walsh et al. (2022) on ASRs living in South Australia on temporary visa shows that “long periods on short-term visas […] without work rights” contribute to precarity and poorer mental health.

Two studies beyond the Australian context support these findings. The mixed-method study of Bjertrup et al. (2018) in Greece reveals that the uncertainty stemming from the complex asylum procedure and the social marginalization caused by the denial of basic rights and services lead to psychological distress and social suffering among ASRs. Llewellyn (2021) showed that work restrictions and shifting timelines cause distress, isolation, prolonged uncertainty, and physical vulnerability among LGBTQ asylum seekers in the US.

#### 3.3.5 Asylum procedure

Five of the reviewed studies report on the direct impact of the asylum procedure – or specific elements of it, such as the length of the asylum process or the asylum interview – on asylum seekers’ mental health. All five studies in this domain uncover negative effects of the asylum procedure on ASRs’ mental health. That said, two of the studies draw a more ambiguous picture and reveal ways in which the asylum procedure can eventually have an overall positive impact on ASRs’ mental health.

The study of Laban et al. (2004) focuses explicitly on the effects of a long asylum procedure. The authors substantiate the link between the protraction of the asylum procedure and the worsening of psychiatric morbidity, attributing the observed results to the uncertainty and the consequent lack of purposeful activities or forward planning. Their findings unequivocally show that elongated timelines in the asylum adjudication processes significantly exacerbate mental health disturbances in ASRs, surpassing the psychological impacts of pre-migration adversities.

Three of the studies in this domain address the mental health impacts of the asylum interview as a pivotal component of the asylum adjudication process. Using different approaches and focusing on different contexts, Chaffelson et al. (2023) and Schock et al. (2015) conclude that asylum interviews exacerbate mental health issues amongst ASRs. In terms of mechanism, Chaffelson et al. (2023) point to the re-traumatization inherent in recounting traumatic experiences and the pervasive atmosphere of skepticism within the UK asylum system. Another study conducted in Canada with LGBTQ asylum claimants shows that disclosure requirements as part of the interview process in combination with compressed timelines in the asylum procedure cause distress and exacerbate existing mental health issues (Kahn and Alessi, 2018). At the same time, it is important to note that two of the abovementioned three studies also reveal some positive effects of the asylum procedure as a whole on claimants’ mental health. More specifically, Chaffelson et al. (2023) show that despite the negative impact of the asylum interview, some asylum seekers may ultimately feel empowered by their overall asylum procedure experience. Relying on service providers’ perspective, Kahn and Alessi (2018) also show that in some cases the refugee claim process in Canada can ultimately empower asylum claimants.

Lastly, the study of Caroppo et al. (2021) examines the impact of procedural policies on mental health focusing on asylum seekers returned to Italy under the Dublin III Regulation. The study introduces a psychopathological condition, the “Dublin Migration Syndrome”, which consists of “the absence of a safe and clear legal status, the threat to the survival instincts, the loss of the migratory project and the alienation mediated by a chronic social defeat paradigm”(Caroppo et al., 2021). This syndrome – characterized by severe mental health conditions such as PTSD, dissociative symptoms, and a broader identity crisis – underscores the profound psychological toll of a provision that leaves individuals in a state of legal limbo.

#### 3.3.6 Asylum procedure and reception

Three studies examined the impact of asylum procedures *in conjunction with* reception policies on ASRs’ mental health, all of them revealing adverse outcomes.

In their study of asylum seekers’ experiences in Sweden, Arwidson et al. (2022) show that the uncertainty and inactivity during extended periods of waiting in collective accommodation centers leads to “intense feelings of psychological distress”, which manifests itself in ruminative thoughts, such as constant worrying and overthinking. In a similar vein, in the Belgium context, Whitehouse et al. (2021) show how poor conditions in reception centers (e.g., absence of privacy, overcrowding, unhygienic conditions), lack of meaningful activities, inadequate provision of psychological support, and overly bureaucratic and administrative procedures contributed to deteriorated mental health. Lastly, the study of Eleftherakos et al. (2018) on the impact of the EU-Turkey deal on asylum seekers on Lesvos provides even more striking evidence. The authors classify the broader reception conditions and asylum procedure on the Greek island as a form of systemic or institutional abuse, characterized by absence of security, intimidation, victimization, inhuman living conditions, uncertainty, and lack of information. As a direct consequence of this, asylum seekers on Lesvos were found to live under continuous traumatic stress: a state of permanent emergency, preoccupation with threats, and absence of protective measures (Eleftherakos et al., 2018).

#### 3.3.7 Reception

Three studies focused exclusively on the direct impact of reception policies on ASRs’ mental health. Echoing the abovementioned findings of Eleftherakos et al. (2018), Pérez-Sales et al. (2022) argue that Moria camp on Lesvos constitutes a “torturing environment” with inhumane living conditions and high level of exposure to violence causing mental health deterioration. In the American context, Mercado et al. (2022) examine the US Migration Protection Protocol (MPP), which forces asylum seekers to wait in Mexico for their asylum claims to be processed. The authors use a mixed-method approach and focus on a tent encampment along the Rio Grande, highlighting the inadequate, unsanitary, and hazardous conditions there. As a result, asylum seekers residing in the encampment were exposed to trauma and suffered from psychological distress, reporting feelings of despair, hopelessness, fear, and severe sleep deprivation, among other symptoms. Lastly, in an arguably less extreme environment, Watters et al. (2022) explore the experiences of resettled refugees in Ireland through a psychological lens. Their findings show that prolonged stay in temporary accommodation centers under the Irish Refugee and Protection Programme, coupled with uncertainty and idleness, led to deteriorated psychological well-being expressed through feelings of helplessness and anxiety.

#### 3.3.8 Access to basic rights, asylum procedure and reception

The last cluster we identified includes three studies that delve into the experiences of ASRs in Norway and Germany to examine the cumulative impact of access to basic rights, asylum procedure, and reception policies on their mental health.

Based on ethnographic fieldwork with asylum seeking women living in reception centers, Willmann-Robleda (2022) concludes that the Norwegian asylum and reception system leads to frustration, apathy, and depression. More specifically, the author identifies five main elements that cause these psychological challenges in asylum seeking women: “the uncertainty around their asylum application coupled with the inability to influence their circumstances, limitations to engage in meaningful activities as well as the financial and mobility limitations imposed by the Norwegian authorities” (Willmann-Robleda, 2022). In their study on experiences of rejected asylum seekers, Sagbakken et al. (2022) reach similar conclusions. The authors show how excessively long asylum procedures, gradual economic and social restrictions, and limited access to healthcare create an environment that perpetuates uncertainty and dehumanization. This environment in turn leads to pervasive feelings of hopelessness and powerlessness among asylum seekers, with the protraction of the asylum procedures further worsening psychiatric morbidity due to the uncertainty and resulting lack of purposeful activities or forward planning. In a similar vein, the study of Scott (2018) shows that rejected Black African asylum seekers in Germany who live on a “Duldung” (a tolerated stay with suspension of deportation) experience stressors of shared accommodation in remote shelters, constraints to basic needs such as food and healthcare, and insecurity and bureaucratic harassment that can lead to psychological distress and mental health problems.

## 4 Findings and recommendations

It has been well established that ASRs display poorer mental health compared to the general population of host countries (Bogic et al., 2015). However, the evidence on the causal impact of legal and policy frameworks as a driver of mental health distress or improvements in ASRs’ after their arrival has not been previously synthesized. Our review shows that the available scholarly research on the impact of Western governments’ laws and policies on ASRs’ mental health is almost exclusively focused on restrictive or deterrence measures. In fact, a number of studies included in our review explicitly framed the laws and policies they examined as based on “deterrence” logic (Bjertrup et al., 2018; Fleay and Hartley, 2016; Targarona Rifa and Donà, 2021; Walsh et al., 2022). This is hardly surprising, as it matches the evident “war on migration” zeitgeist in the West since the 1990s (De Haas, 2023; Webber, 2004). Expectedly, therefore, our synthesis shows that Western governments’ laws and policies in this period have overwhelmingly had a negative impact on ASRs’ mental health. This negative impact is clearly traceable across different legal and policy domains that regulate ASRs’ detention, reception, processing, and access to basic rights.

Although our findings are focused exclusively on arguments for the causal link between Western governments’ legal/policy measures and negative mental health impacts among ASRs’, it is important to note that they are also in line with the findings of numerous studies with broader focus. For example, a large body of cross-sectional studies has highlighted the irrefutable association between visa insecurity and adverse mental health impacts (Boettcher and Neuner, 2022; Newnham et al., 2019; Nickerson et al., 2019; Ryan et al., 2009), or long asylum waiting times and mental health deterioration (Hvidtfeldt et al., 2020; Phillimore and Cheung, 2021). Moreover, our findings are also in line with the conclusions of previous systematic reviews with broader scope, which have highlighted the negative mental health impacts of detention across different locations (von Werthern et al., 2018), as well as the negative mental health impacts of restrictive entry and welfare policies for immigrants (Juárez et al., 2019).

Our review also gives some insights into the underlying mechanisms that explain the negative effect of specific laws and policies on ASRs’ mental health. In this respect, our synthesis elucidates first and foremost the importance of uncertainty. Although an inherent part of every refugee experience, post-arrival uncertainty seems to be commonly exacerbated by Western governments’ legal and policy measures, especially in the domain of detention and asylum procedure. Paramount in this respect is the pervasive uncertainty stemming from detention with no clarity for the detained subjects on their future or eventual release date (Cleveland et al., 2018; Hollis, 2019). Other common explanatory mechanisms that link laws/policies and ASRs’ deteriorating mental health according to our review are social isolation (stemming from laws/policies regulating reception and access to basic rights) and dehumanization (detention and reception).

At the same time, and in contrast to the above, it is also important to acknowledge the evidence for the positive impact of some Western government policies on ASRs’ mental health. Although very limited, this evidence shows that there are ways to mitigate the negative effect of pre-, peri‑, and post-migration stressors by facilitating ASRs’ early and easy access to healthcare and psychological support services (Hocking et al., 2015; Jaschke and Kosyakova, 2021).

The latter point brings us to our first recommendation directed to scholarly audiences. It may appear sensible or even desirable to focus on the “dark side” of laws and policies in relation to their mental health consequences. That said, it is equally important to also study examples of governmental measures that are likely to have positive impacts on ASRs’ mental health. In this respect, there are a number of research avenues that are worth exploring. One example would be policies that provide reception in individual/family units (e.g., apartments in urban and rural areas) rather than in collective accommodation centers, along with provision of socio-psychological support and other services. Such policies have operated in different countries, including Italy and Greece. Another example would be refugee sponsorship programs, which facilitate accommodation in private housing and tailored support by ordinary individuals (including family members in some cases) for a period from several months to up to two years (Labman and Cameron, 2020). Lastly, one more suggestion to trace positive mental health impacts of Western governments’ laws and policies would be the implementation of the Temporary Protection Directive in the EU for individuals fleeing Ukraine, which facilitated immediate access to rights and services.

Another scholarly recommendation that emerges from our analysis relates to the apparent gaps in conceptualizing mental health. As discussed above, only eight of the reviewed 34 studies offered an explicit definition of mental health and subsequently justified their choice of mental health measures based on this definition. Beyond the already mentioned validity concerns, the lack of engagement with the concept of mental health in research on the impact of migration-related laws and policies hides additional risks. For example, it may result in overlooking the importance of developing culturally informed mental health conceptualizations aligned with one’s research setting and participants, or it may result in privileging/perpetuating Western perspectives on mental health with all corresponding consequences for ASRs’ from very different backgrounds. Therefore, we encourage researchers to consider engaging explicitly with the conceptualization of mental health in future research focusing on the impact of law and policies for ASRs.

Finally, based on our findings we also offer a recommendation for legislators and policymakers. Our synthesis suggests that Western governments’ shift towards deterrence-oriented laws and policies has been taking a heavy toll on the mental health of asylum seekers and refugees. What is more, our synthesis also shows that the harmful effects are not temporally restricted to the period in which ASRs are subjected to these laws and policies. Importantly, the majority of ASRs remain in their country of arrival and eventually settle there, including both those who manage to secure permanent residence status and those who are eventually denied protection. In this respect, it is essential to consider the long-term consequences and costs of deterrence measures through the prism of mental health. Although largely invisible – or perhaps more accurately, “invisibilized” – these consequences and costs should be a warning signal for the urgent need to introduce more humane measures that provide certainty and opportunities for developing social relations and rebuilding one’s life.

## 5 Limitations

In closing, we acknowledge that our study has several notable limitations (in addition to the limitations stemming from the lack of mental health definition in most of the reviewed studies, as discussed above). Despite our broad search strategy including six databases, multiple search terms, and hand search, we may still have failed to screen all available studies on the impact of Western governments’ laws and policies on ASR’s mental health. Searching additional databases and extending the list of relevant search terms may help remedy this shortcoming. Moreover, our review was restricted to English-language studies. Extending it to publications in other languages may well bring additional evidence that could help refine the above synthesis. Lastly, we acknowledge that our exclusive focus on the *causal* impact of laws and policies on ASRs’ mental health inevitably results in a certain dose of subjectivity in the study selection process. We have attempted to tackle this issue by providing a detailed account of the reasons for excluding studies that in our view do not present a compelling causal argument (Appendix B). Notwithstanding these limitations, we argue that our systematic-narrative hybrid literature review offers a unique insight into the current evidence base on the impact of laws and policies on the mental health of ASRs in Western countries.

## CRediT authorship contribution statement

**Imen El Amouri:** Writing – review & editing, Writing – original draft, Visualization, Validation, Methodology, Investigation, Formal analysis, Data curation, Conceptualization. **Tihomir Sabchev:** Writing – review & editing, Writing – original draft, Validation, Supervision, Methodology, Investigation, Formal analysis, Data curation, Conceptualization.

## Declaration of competing interest

The authors declare the following financial interests/personal relationships which may be considered as potential competing interests: Imen El Amouri reports financial support was provided by Foundation for the Support Association for Christian Care of the Mentally ill. If there are other authors, they declare that they have no known competing financial interests or personal relationships that could have appeared to influence the work reported in this paper.

## Figures and Tables

**Table:** 

Study	Exclusion criteria	Explanation
Ali-Hassan, Y. et al. (2021) ‘Variations in perceived stress among Syrian refugee parents resettled through different sponsorship programs in Canada’, Journal of Migration and Health, 4: 1–6. https://doi.org/10.1016/j.jmh.2021.100066.	The authors explicitly state that causal claims cannot be made on the basis of their study.	"Perceived stress score" is the main outcome that the authors measure. The main independent variables are the different sponsorship programs and covariates (demographic, economic, social factors). The study shows that the type of sponsorship program (PSR, GAR, BVOR) is a significant predictor of perceived stress after adjusting for several demographic, economic, and social factors. The authors acknowledge that the reason behind the poor stress outcomes for BVOR is "not fully understood and difficult to explain". They provide some suggestions for possible explanations based on previous literature on the sponsorship programs, but do not make any causal claims. The authors also acknowledge in their limitations that the direction of causality between perceived stress score and sponsorship program is not clear, and their results are subject to confounding biases. Importantly, the authors do not study specific elements of each program and their relationship with perceived stress score.
Bakker, L., Dagevos, J., Engbersen, G. (2014) ‘The Importance of Resources and Security in the Socio-Economic Integration of Refugees. A Study on the Impact of Length of Stay in Asylum Accommodation and Residence Status on Socio-Economic Integration for the Four Largest Refugee Groups in the Netherlan’, Journal of International Migration and Integration, 15/3: 431–48. https://doi.org/10.1007/s12134–013–0296–2.	The authors explicitly state that causal claims cannot be made on the basis of their study.	The authors explore length of stay in asylum accommodation and residence status, their impact on refugees' mental health, and the subsequent impact on refugee socio-economic integration (employment status, occupational status, and social benefits dependency). The main focus is on the relationship between policy (length of stay in asylum accommodation & residence status) and socio-economic integration, while mental health is studied as a mediator (a personal resource that is affected by the policy and that would impact integration). The authors acknowledge that their results are reported as associations because they could not test the direction of relations between dependent and independent variables due to the cross-sectional nature of their data. As the authors mention themselves, "it is not unlikely that an inverse relationship between socio-economic integration and mental health exists as well".
Bhatia, M. (2020) ‘Crimmigration, imprisonment and racist violence: Narratives of people seeking asylum in Great Britain’, *Journal of Sociology*, 56/1: 36–52. https://doi.org/10.1177/1440783319882533.	The explored link is not between specific laws/policies and mental health impacts.	Rather than on specific laws/policies, the focus of the article is on the impact of actions and inactions of street-level bureaucrats across different policy domains, and the way these actions and inactions can harm asylum seekers (within and beyond the mental health realm). For example, the Home Office or housing providers ignoring relocation requests from asylum seekers, the Police ignoring complaints from asylum seekers, GPs not accepting asylum seeker patients, or not arranging interpreters for them, etc.
Bloom, A., Udahemuka, M. (2014) ‘‘Going through the doors of pain’: asylum seeker and Convention refugee experiences in Aotearoa New Zealand’, Kotuitui, 9/2: 70–81. https://doi.org/10.1080/1177083X.2014.939664.	The authors acknowledge limitations and do not present their results in terms of a causal link between law/policy and ASRs' mental health.	The authors admit that their research "is not intended to provide a detailed analysis of the effects of policy on asylum seekers and Convention refugees". In this respect, the article outlines the context by "summarizing some of the policies that present challenges for asylum seekers and Convention refugees". Furthermore, the authors acknowledge that their findings cannot be considered to be representative for the entire population of Convention refugees in Aotearoa, New Zealand. Although there is some evidence linking periods of prolonged uncertainty and deteriorating mental health, the authors do not frame this as a causal link between the existing policy and asylum seekers' mental health outcomes.
Boettcher and Neuner (2022) ‘The Impact of an Insecure Asylum Status on Mental Health of Adult Refugees in Germany’, Clin Psychol Eur, 4/1: e6587-e87. https://doi.org/10.32872/cpe.6587.	The authors explicitly state that causal claims cannot be made on the basis of their study.	The study explores "the additive effect of insecure status on PTSD symptomatology in refugees, over and above the influence of other pre-and peri‑migration factors". It looks at several different statuses that forced migrants may hold after asking for asylum in Germany. The findings show that "people with an insecure asylum status are at higher risk for an increased PTSD symptomatology". The authors acknowledge that their study does not investigate causation, but only provides strong evidence for the association between the variables (despite relying on convenience samples, rather than on random selection).
Brekke, J.-P. (2010) ‘Life on hold: the impact of time on young asylum seekers waiting for a decision’, Diskurs Kindheits- und Jugendforschung / Discourse Journal of Childhood and Adolescence Research, 5/2: 159–67.	The explored link is not between specific laws/policies and mental health impacts.	The author explores the uncertainty inherent in the asylum seeking process (uncertainty about the process itself as well as the outcome of it) and its impact on asylum seekers' mental health, rather than the causal link between a specific policy and mental health outcomes.
Choummanivong, C., Poole, G. E., Cooper, A. (2014) ‘Refugee family reunification and mental health in resettlement’, Kotuitui, 9/2: 89–100. https://doi.org/10.1080/1177083X.2014.944917.	The explored link is not between specific laws/policies and mental health impacts.	The authors focus on the impact of family reunification on mental health and resettlement outcomes of refugees. The mental health impacts are overwhelmingly examined as a function of the family reunification itself and its dynamics (family separation, intergenerational conflicts, rejection of the application for family reunification, etc.) rather than a function of the state policy on family reunification.
Chung, R. C. Y., Bemak, F. (1996) ‘The effects of welfare status on psychological distress among Southeast Asian refugees’, Journal of Nervous and Mental Disease, 184/6: 346–53. https://doi.org/10.1097/00005053–199606000–00003.	The explored link is not between specific laws/policies and mental health impacts.	The article examines the impact of welfare dependency on the mental health of refugees. Rather than focusing on the welfare policy and its elements, it establishes a link between welfare status (being on welfare or not) and mental health of individuals.
Comtesse, H., Rosner, R. (2019) ‘Prolonged grief disorder among asylum seekers in Germany: the influence of losses and residence status’, Eur J Psychotraumatol, 10/1: 1591,330–30. https://doi.org/10.1080/20008198.2019.1591330.	The explored link is not between specific laws/policies and mental health impacts.	The study examines the risk factors of prolonged grief disorder (PGD) in asylum seekers at different stages of the asylum procedure. The evidence shows an association between residence permit status and lower PGD symptom levels. The authors do not "unpack" the meaning of status in terms of access to different rights and services. They put forward some suggestions about what might explain the association they find, but do not make any causal claims.
Damen, R. E. C., Dagevos, J., Huijnk, W. (2022) ‘Refugee Reception *Re*-examined: a Quantitative Study on the Impact of the Reception Period for Mental Health and Host Country Language Proficiency Among Syrian Refugees in the Netherlands’, Journal of International Migration and Integration, 23/1: 1–21. https://doi.org/10.1007/s12134–021–00820–6.	The authors acknowledge limitations and do not present their results in terms of a causal link between law/policy and ASRs' mental health.	The study examines the impact of the Dutch reception policy (including length of stay in a reception centre, relocations between reception centres, and activities in the reception centre) on refugees’ mental health. The authors do not find a relationship between length of stay in a reception centre and mental health, but does find that the number of relocations is negatively related to refugee mental health, and activities are positively related to refugee mental health. The lack of data on the level of pre-arrival trauma, the cross-sectional nature of the data, and the possibility that some of the observed relations can be reciprocal, limits the findings of the authors to associations, and they do not engage in making any causal claims.
Drozdek, B. et al. (2013) ‘Is legal status impacting outcomes of group therapy for posttraumatic stress disorder with male asylum seekers and refugees from Iran and Afghanistan?’, Bmc Psychiatry, 13: 10–10. https://doi.org/10.1186/1471–244x–13–148.	The explored link is not between specific laws/policies and mental health impacts.	The study examines "whether legal status and other resettlement stressors influence outcomes of a trauma-focused group PTSD treatment within a day-treatment setting with asylum seekers and refugees". The findings show that being granted a permanent refugee status leads to reduction in anxiety, depression, and PTSD symptoms. The authors do not "unpack" the meaning of status though, nor any policy associated with it.
Dudek, V., Razum, O., Sauzet, O. (2022) ‘Association between housing and health of refugees and asylum seekers in Germany: explorative cluster and mixed model analysis’, Bmc Public Health, 22/1. https://doi.org/10.1186/s12889–021–12458–1.	The authors explicitly state that causal claims cannot be made on the basis of their study.	The authors examine the mental health impact of refugee accommodation. By refugee accommodation, the authors only partially refer to the relevant laws and policies. They look therefore at relationships between different "housing conditions clusters" on the one hand and mental health on the other. Furthermore, the authors explicitly acknowledge that their study design (cross-sectional) does not allow conclusions on causality of the identified associations.
Garton, A., Rogers, K., Berle, D. (2022) ‘Reciprocal relationships between employment status and psychological symptoms: findings from the Building a New Life in Australia study’, Social Psychiatry and Psychiatric Epidemiology, 57/5: 1085–95. https://doi.org/10.1007/s00127–021–02204–8.	The explored link is not between specific laws/policies and mental health impacts.	The article looks at the link between employment status (working or not being in paid work) and symptoms of PTSD and psychological distress. The authors find that the variables are indeed correlated, and they reciprocally influence each other over time. However, the study is not related to a specific policy per se, but rather to the employment status of refugees.
Griswold, K. S. et al. (2021) ‘"I just need to be with my family": resettlement experiences of asylum seeker and refugee survivors of torture’, Globalization and Health, 17/1: 7–7. https://doi.org/10.1186/s12992–021–00681–9.	The explored link is not between specific laws/policies and mental health impacts.	The study explores the experiences of survivors of torture (self-identified) living in New York. It identifies a lot of mental health challenges linked to previous trauma and concerns about family overseas. Although some psychological challenges are related to post-migration stressors, the authors do not clearly link them to specific policies.
Guajardo, M. G. U. et al. (2016) ‘Psychological distress is influenced by length of stay in resettled Iraqi refugees in Australia’, International Journal of Mental Health Systems, 10: 7–7. https://doi.org/10.1186/s13033–016–0036-z.	The explored link is not between specific laws/policies and mental health impacts.	The article examines the influence of length of stay in Australia on the psychological distress of Iraqi refugees. The results show that refugees with longer periods of resettlement are experiencing higher levels of psychological distress.
Hallas, P. et al. (2007) ‘Length of stay in asylum centres and mental health in asylum seekers: A retrospective study from Denmark’, Bmc Public Health, 7. https://doi.org/10.1186/1471–2458–7–288.	The authors acknowledge limitations and do not present their results in terms of a causal link between law/policy and ASRs' mental health.	The study provides evidence that referrals for mental disorders (psychiatric diagnosis, somatic diagnosis, mental illness) increase with length of stay in Danish asylum centres. The authors present their findings in terms of association between the variables, and do not make causal claims.
Hedrick, K. (2017) ‘Getting out of (self-) harm's way: A study of factors associated with self-harm among asylum seekers in Australian immigration detention’, Journal of Forensic and Legal Medicine, 49: 89–93. https://doi.org/10.1016/j.jflm.2017.05.014.	The author acknowledges limitations and does not present the results in terms of a causal link between law/policy and ASRs' mental health.	The study presents a descriptive analysis of self-harm incident reports from Australian immigration detention centres. It describes methods used to self-harm and precipitating factors for self-harm. The study identifies several such precipitating factors for self-harm, including detention conditions and processing arrangements, but does not make causal claims.
Heeren, M. et al. (2014) ‘Psychopathology and resident status - Comparing asylum seekers, refugees, illegal migrants, labor migrants, and residents’, Comprehensive Psychiatry, 55/4: 818–25. https://doi.org/10.1016/j.comppsych.2014.02.003.	The authors explicitly state that causal claims cannot be made on the basis of their study.	The study describes and compares mental health outcomes in four different migrant groups in Switzerland. The results show an association between status (e.g., asylum seeker, refugee, etc.) and psychopathology. The data of the authors is cross-sectional, and they acknowledge that it is not possible to make causal claims based on it.
Hess, J. M. et al. (2019) ‘Refugee Mental Health and Healing: Understanding the Impact of Policies of Rapid Economic Self-sufficiency and the Importance of Meaningful Work’, Journal of International Migration and Integration, 20/3: 769–86. https://doi.org/10.1007/s12134–018–0628–3.	The explored link is not between specific laws/policies and mental health impacts.	The study explores culturally specific perspectives on trauma and recovery among refugees resettled in the US. The findings highlight the importance of productivity (work, but also language and skills) for recovery from post trauma. Based on the significance that refugees put on productivity, the authors make some policy recommendations, but they do not explore causal links between specific policies and refugees' mental health in their analysis.
Hourani, J. et al. (2022) ‘How structural and symbolic violence during resettlement impacts the social and mental wellbeing of forced migrant women: the lived experiences of Arabic speaking survivors of IPV resettled in Melbourne, Australia’, Conflict and Health, 16/1: 9–9. https://doi.org/10.1186/s13031–022–00494–6.	The explored link is not between specific laws/policies and mental health impacts.	The study explores different forms of violence (structural, symbolic, interpersonal) and their impact on the mental health of forced migrants in Australia. The findings highlight the importance of autonomy and independence - broadly speaking, rather than in relation to a result of specific policy - for the mental health of forced migrant women.
Hvidtfeldt et al. (2020) ‘Prolonged periods of waiting for an asylum decision and the risk of psychiatric diagnoses: a 22-year longitudinal cohort study from Denmark’, International Journal of Epidemiology, 49/2: 400–09. https://doi.org/10.1093/ije/dyz091.	The authors explicitly state that causal claims cannot be made on the basis of their study.	The study explores whether long asylum-decision waiting periods are associated with the risk of being diagnosed with a psychiatric disorder among resettled refugees in Denmark. The findings show a positive association between the two variables. The authors acknowledge that they are not able to make causal claims based on their data (the direction of the cause-effect relationship not being clear).
Hvidtfeldt, C., Petersen, J. H., Norredam, M. (2022) ‘Waiting for family reunification and the risk of mental disorders among refugee fathers: a 24-year longitudinal cohort study from Denmark’, Soc Psychiatry Psychiatr Epidemiol, 57/5: 1061–72. https://doi.org/10.1007/s00127–021–02170–1.	The authors explicitly state that causal claims cannot be made on the basis of their study.	The study examines whether family separation is associated with the risk of mental disorders among refugee fathers in Denmark. It finds that the risk of mental disorders increases with longer periods of waiting for family reunification. The authors acknowledge that they are not able to establish a causal relationship because they cannot establish variation in data unrelated to their exposure variables (e.g., legal amendments).
Isaacs, A. et al. (2022) ‘‘I don’t think there’s anything I can do which can keep me healthy’: how the UK immigration and asylum system shapes the health & wellbeing of refugees and asylum seekers in Scotland’, Critical Public Health, 32/3: 422–32. https://doi.org/10.1080/09581596.2020.1853058.	The explored link is not between specific laws/policies and mental health impacts.	The study examines the experiences of structural violence of asylum seekers and refugees from Sub-Saharan Africa in Glasgow. The authors argue that the structure of the asylum system makes individuals vulnerable to physical and mental health, but do not claim that the former in terms of specific policy/ies is causing mental health deterioration.
Kleinert, E. et al. (2019) ‘Does refugee status matter? Medical needs of newly arrived asylum seekers and resettlement refugees-a retrospective observational study of diagnoses in a primary care setting’, Conflict and Health, 13/1: 9–9. https://doi.org/10.1186/s13031–019–0223-z.	The authors acknowledge limitations and do not present their results in terms of a causal link between law/policy and ASRs' mental health.	The study explores the mental health needs of asylum seekers and refugees in Germany. Results show that asylum seekers present more often mental and behavioural disorders compared to resettled refugees. The authors do not make any causal claims linking laws or policies with the higher prevalence of mental and behavioural disorders among asylum seekers.
Koehn, P. H. (2006) ‘Transnational migration, state policy and local clinician treatment of asylum seekers and resettled migrants: Comparative perspectives on reception center and community health care practice in Finland’, Global Social Policy, 6/1: 21–56. https://doi.org/10.1177/1468018106061391.	The authors acknowledge limitations and do not present their results in terms of a causal link between law/policy and ASRs' mental health.	The study compares asylum seekers and foreign-born residents in Finland in relation to their health care treatment and outcome perspectives. The results show that asylum seekers with mental health problems believe that their mental health would improve if they were given legal residence in Finland. However, the authors do not make any claims for a causal link between asylum seeker status and mental health problems.
Lamkaddem, M. et al. (2015) ‘Health changes of refugees from Afghanistan, Iran and Somalia: the role of residence status and experienced living difficulties in the resettlement process’, European Journal of Public Health, 25/6: 917–22. https://doi.org/10.1093/eurpub/ckv061.	The explored link is not between specific laws/policies and mental health impacts.	The study explores the impact of obtaining residence permit on asylum seekers' mental and physical health in the Netherlands. The results show that the transition from asylum seeker to permit holder is associated with improved mental health. The association is mediated by the change in living conditions that individuals experience in this transition. The authors do not elaborate on the relevance of policies in this link and define "living conditions" rather loosely, with focus on employment, presence of family/social support.
Lintner, C., Elsen, S. (2018) ‘Getting out of the seclusion trap? Work as meaningful occupation for the subjective well-being of asylum seekers in South Tyrol, Italy’, Journal of Occupational Science, 25/1: 76–86. https://doi.org/10.1080/14427591.2017.1373256.	The explored link is not between specific laws/policies and mental health impacts.	The study focuses on the lack of valued and meaningful occupation during the asylum seeking process in South Tyrol, Italy, as a cause of deteriorating mental health. However, this lack of meaningful occupation is not examined as a consequence of a policy (all asylum seekers interviewed for the study had the right to work).
Löbel, L. M., Jacobsen, J. (2021) ‘Waiting for kin: a longitudinal study of family reunification and refugee mental health in Germany’, Journal of Ethnic and Migration Studies, 47/13: 2916–37. https://doi.org/10.1080/1369183X.2021.1884538.	The explored link is not between specific laws/policies and mental health impacts.	The study explores the association between family reunification and mental health of ASRs in Germany. The authors look at both family reunification through institutional channels and self-navigated reunifications (subsequent arrival of a family member to Germany by own means). The authors acknowledge that their study is not a policy analysis and note that the link between family reunification policy and mental health is worth studying in future research.
Mahmood, A. A. et al. (2020) ‘Assessing Predictors of Emotional Distress by Immigrant Type: An Exploration of Adult Refugees, Asylees, and SIV Holders in Maryland’, Journal of Immigrant and Minority Health, 22/1: 50–60. https://doi.org/10.1007/s10903–019–00917–2.	The explored link is not between specific laws/policies and mental health impacts.	The study explores predictors of elevated distress among refugees, asylees, and Special Immigrant Visa Holders in Maryland, US. The findings show elevated stress from some groups/demographics. The authors admit that the extent to which this elevated distress can be attributed to experiences specific to the immigrant type is a limitation of their study.
Moreira, A. L. et al. (2020) ‘Lives on hold: The experiences of asylum seekers in moria refugee camp’, Community Psychology in Global Perspective, 6/1: 92–107.	The authors acknowledge limitations and do not present their results in terms of a causal link between law/policy and ASRs' mental health.	The study describes the lived experiences of asylum seekers residing in Moria and Kara Tepe camps in Greece, and the way these experiences affect asylum seekers' psychological wellbeing. The authors provide a lot of evidence of the adverse living conditions in the camps. However, when it comes to mental health impacts, the empirical evidence is overwhelmingly focused on the negative impact of traumatic events in the pre- and peri‑migration period, rather than the Greek reception policy.
Mueller, J. et al. (2011) ‘Mental health of failed asylum seekers as compared with pending and temporarily accepted asylum seekers’, European Journal of Public Health, 21/2: 184–89. https://doi.org/10.1093/eurpub/ckq016.	The authors explicitly state that causal claims cannot be made on the basis of their study.	The study explores the mental health of failed asylum seekers in comparison to the mental health of asylum seekers in Switzerland. The results show severe mental health problems in both samples, related to post-migration living difficulties. The authors acknowledge that the cross-sectional design of their study does not allow them to make causal inferences.
Murphy, R., Keogh, B., Higgins, A. (2021) ‘An embodied distress: African asylum seekers’ experiences of mental health difficulties while awaiting an asylum outcome in Ireland’, Transcultural Psychiatry, 58/2: 239–53. https://doi.org/10.1177/1363461520966108.	The authors acknowledge limitations and do not present their results in terms of a causal link between law/policy and ASRs' mental health.	The study explores African asylum seekers' experiences of mental distress in Ireland. The authors provide some evidence on the impact of the Irish Direct Provision system of reception on asylum seekers' mental health, but only in combination with the impact of pre-migratory traumas and feelings of self-blame. The possible causal impact of the Direct Provision on asylum seekers' mental health is therefore not evidenced.
Newnham et al. (2019) ‘The mental health effects of visa insecurity for refugees and people seeking asylum: a latent class analysis’, International Journal of Public Health, 64/5: 763–72. https://doi.org/10.1007/s00038–019–01249–6.	The authors explicitly state that causal claims cannot be made on the basis of their study.	The study examines the relationship between visa insecurity and psychological outcomes of asylum seekers and refugees in Australia. The results show that people with insecure visa status are much more likely to report high or very high depression and anxiety symptoms compared to those with permanent residency. The authors acknowledge that they are unable to infer causality due to the cross-sectional nature of their data.
Nickerson et al. (2019) ‘The association between visa insecurity and mental health, disability and social engagement in refugees living in Australia’, European Journal of Psychotraumatology, 10/1: 1688,129. https://doi.org/10.1080/20008198.2019.1688129.	The explored link is not between specific laws/policies and mental health impacts.	The study explores the association between insecure visa status and mental health of refugees and asylum seekers in Australia. Rather than focusing on specific policy, the authors combine under insecure status very different arrangements (temporary protection visa, expired visa, no visa). The same goes for secure visa status (permanent residency or Australian citizenship). In addition, the authors note that longitudinal data is needed to determine the direction of causality (although without specifically admitting that their inference is not causal due to the cross-sectional data they have).
Pernice, R., Brook, J. (1994) ‘Relationship of migrant status (refugee or immigrant) to mental health’, International Journal of Social Psychiatry, 40/3: 177–88. https://doi.org/10.1177/002076409404000303.	The explored link is not between specific laws/policies and mental health impacts.	The study explores and compares mental health levels among refugees and other categories of immigrants living in New Zealand. The hypothesis that migrant status (being a refugee or immigrant) affects mental health and that refugees experience higher level of emotional distress is only partially supported by the authors' findings. However, the study does not inquire into the pre-migration experiences of the participants and also does not unpack the meaning of status in terms of legal/policy provisions.
Phillimore and Cheung (2021) ‘The violence of uncertainty: Empirical evidence on how asylum waiting time undermines refugee health’, Social Science & Medicine, 282. https://doi.org/10.1016/j.socscimed.2021.114154.	The authors acknowledge limitations and do not present their results in terms of a causal link between law/policy and ASRs' mental health.	The study explores the impact of uncertainty in the form of asylum waiting times on refugees' self-reported health. The findings show that lengthy asylum waiting times are associated with physical and emotional harms. The authors describe their findings in terms of association, rather than causal relationship and the study also has several limitations (sample attrition, not unpacking the policy but only considering waiting time, not considering the level of pre-arrival trauma, focus on emotional, rather than psychological health, etc.).
Rees, S. (2003) ‘Refuge or retrauma? The impact of asylum seeker status on the wellbeing of East Timorese women asylum seekers residing in the Australian community’, Australasian Psychiatry, 11/Suppl1: S96-S101. https://doi.org/10.1046/j.1038–5282.2003.02022.x.	The author acknowledges limitations and does not present the results in terms of a causal link between law/policy and ASRs' mental health.	The study relies on interviews with East Timorese asylum seeker women and questionnaires with service providers in Australia to examine the effects of prolonged asylum seeker status on wellbeing, with the latter including also psychological welfare. The findings pertaining to the psychological aspects of wellbeing are limited to the importance of insecurity of visa status and the associated fear of removal, as well as the long periods of time waiting for refugee applications to be determined, rather than the policy as a whole.
Ryan, D. A., Benson, C. A., Dooley, B. A. (2008) ‘Psychological distress and the asylum process - A longitudinal study of forced migrants in Ireland’, Journal of Nervous and Mental Disease, 196/1: 37–45. https://doi.org/10.1097/NMD.0b013e31815fa51c.	The authors explicitly state that causal claims cannot be made on the basis of their study.	The study examines the relationship between legal status security and psychological distress of forced migrants in Ireland. The results show that insecure legal status is associated with higher distress. The authors acknowledge that the correlational nature of their data makes it impossible to make causal inferences.
Rzepka, I. et al. (2022) ‘Impact of and Coping with Post-Traumatic Symptoms of Refugees in Temporary Accommodations in Germany: A Qualitative Analysis’, International Journal of Environmental Research and Public Health, 19/17. https://doi.org/10.3390/ijerph191710893.	The explored link is not between specific laws/policies and mental health impacts.	The study explores individual experiences of the impact of refugees' trauma sequelae symptoms on their current living conditions and interactions. The participants in the study are both asylum seekers and refugees, and the evidence focuses on post-migration stressors and their impact on mental health (legal uncertainty and fear about deportation, concern about family and friends left behind, etc.), rather than specific laws or policy/ies.
Schoretsanitis, G. et al. (2018) ‘Upon rejection: Psychiatric emergencies of failed asylum seekers’, International Journal of Environmental Research and Public Health, 15/7. https://doi.org/10.3390/ijerph15071498.	The explored link is not between specific laws/policies and mental health impacts.	The study examines patients receiving a psychiatric consultation after their asylum application has been rejected, comparing them with asylum seekers with pending applications. Results show that in most cases the negative decision triggers severe psychic symptoms that lead to the psychiatric consultation. The authors posit that negative asylum decisions may aggravate pre-existing symptoms, rather than triggering them.
Shishehgar, S. et al. (2021) ‘A qualitative study of experiences of asylum-seeker women of living in detention centres: confinement versus safety’, Contemporary Nurse, 57/1–2: 51–64. https://doi.org/10.1080/10376178.2021.1927771.	The authors acknowledge limitations and do not present their results in terms of a causal link between law/policy and ASRs' mental health.	The study explores the experiences of asylum seeker women from Iran in an Australian detention centre. It highlights the ambiguous impact of detention: some women felt powerless and had a negative experience overall, while others compared the detention centre to "paradise". The author acknowledges the shortcomings of the study, its exploratory character, and the need to study the relationship between duration of detention and short/long-term psychological harm by "using appropriately designed quantitative studies".
Silove, D. et al. (2007) ‘The impact of the refugee decision on the trajectory of PTSD, anxiety, and depressive symptoms among asylum seekers: a longitudinal study’, Am J Disaster Med, 2/6: 321–29.	The explored link is not between specific laws/policies and mental health impacts.	The study examines the impact of the refugee decision on asylum seekers' mental health in Australia. The results show that post-decision, the accepted refugees show substantial improvement in mental health outcomes, while the rejected group maintains high level of psychiatric symptoms. The study does not unpack the asylum policy, but focuses exclusively on the impact of a positive versus negative decision.
Solberg, O. et al. (2020) ‘Asylum-seekers' psychosocial situation: A diathesis for post-migratory stress and mental health disorders?’, Journal of Psychosomatic Research, 130: 7–7. https://doi.org/10.1016/j.jpsychores.2019.109914.	The authors acknowledge limitations and do not present their results in terms of a causal link between law/policy and ASRs' mental health.	The study explores the association between mental health and early post-migratory stress among asylum seekers in Sweden. The results suggest that asylum seekers' psychosocial situation might be a diathesis that interacts with early post-migratory stressors and thus have detrimental effect on mental health. The authors acknowledge the limitation stemming from the cross-sectional nature of the data and do not present their findings in terms of causal relationships.
Steele, L. S. et al. (2002) ‘The impact of policy changes on the health of recent immigrants and refugees in the inner city - A qualitative study of service providers' perspectives’, Canadian Journal of Public Health-Revue Canadienne De Sante Publique, 93/2: 118–22. https://doi.org/10.1007/bf03404551.	The authors acknowledge limitations and do not present their results in terms of a causal link between law/policy and ASRs' mental health.	The study explores service providers' opinions about the impact of major policy changes on the health of recent immigrants and refugee communities in Toronto, Canada. It is an exploratory and descriptive study that only partially addresses mental health impacts and does not argue for a causal link between specific policy changes and newcomers' mental health.
Sultan, A., O'Sullivan, K. (2001) ‘Psychological disturbances in asylum seekers held in long term detention: a participant-observer account’, Medical Journal of Australia, 175/(11–12): 593–96.	The authors acknowledge limitations and do not present their results in terms of a causal link between law/policy and ASRs' mental health.	Although the article does report on the mental health of detainees in an Australia detention centre, it does not provide evidence for a causal link between detention and mental health of asylum seekers (included rejected ones), besides the "observation" of the authors. Also, there is no efforts to account for the effect of confounding variables in the discussion on mental health outcomes of detained asylum seekers.
Tschalaer, M. (2022) ‘Queer Necropolitics: Experiences of LGBTQI+ Asylum Claimants During Covid-19 in the UK’, Journal of Contemporary European Research, 18/1: 115–32. https://doi.org/10.30950/jcer.v18i1.1262.	The author acknowledges limitations and does not present the results in terms of a causal link between law/policy and ASRs' mental health.	The study examines the UK government approach to providing accommodation and health services to LGBTQI+ asylum seekers during the Covid-19 pandemic. The findings show that LGBTQI+ asylum seekers may feel depressed and even suicidal as a result of the measures imposed during the Covid-19 pandemic, which compound the effect of the general "hostile environment" policy of the UK government towards asylum seekers.
Tschalär, M. (2022) ‘Queering migration temporalities: LGBTQI+ experiences with waiting within Germany’s asylum system’, Ethnic and Racial Studies. https://doi.org/10.1080/01419870.2022.2076566.	The explored link is not between specific laws/policies and mental health impacts.	The study examines LGBTQI+ asylum seekers' experiences with waiting for decision on their application. Waiting is perceived by the author as an inherent part of EU migration policy. The examples that the author provides link mental health impacts to the monotony of everyday life, internalized homophobia, and broader systems of racism and homophobia. The author claims that waiting relates to extreme forms of social isolation, loneliness and vulnerability, which "potentially contributes to mental health challenges". The participants in the research have been waiting for years for their decision, while the average waiting time in Germany - as mentioned by the author - is 6.1 months.
van de Wiel, W. et al. (2021) ‘Mental health consequences of long-term stays in refugee camps: preliminary evidence from Moria’, Bmc Public Health, 21/1: 10–10. https://doi.org/10.1186/s12889–021–11301-x.	The authors explicitly state that causal claims cannot be made on the basis of their study.	The study explores the relationship between the length of stay in Moria camp and the incidence of acute mental health crises among asylum seekers. The results show a positive correlation between the two variables. However, the authors acknowledge that their study does not provide evidence of causality.
van der Linden, M., Weeda, L., Dagevos, J. (2022) ‘The pains and gains of reception centres: How length of stay in reception centres is associated with Syrian refugees' mental health during early resettlement’, International Migration. https://doi.org/10.1111/imig.13052.	The authors explicitly state that causal claims cannot be made on the basis of their study.	The study examines the extent to which the length of stay in reception centres is associated with refugees' mental health in the Netherlands. It shows that the length of stay in a reception centre exerts an indirect influence on mental health via relocations between reception centres. The authors acknowledge the limitations of the study and note that they do not make any causal inferences.
von Lersner, U., Elbert, T., Neuner, F. (2008) ‘Mental health of refugees following state-sponsored repatriation from Germany’, Bmc Psychiatry, 8: 16–16. https://doi.org/10.1186/1471–244x–8–88.	The explored link is not between specific laws/policies and mental health impacts.	The study explores the experiences of individuals participating in state-sponsored repatriation program from Germany. The authors show that traumatic events before or after the return may result in increased mental health problems of the returnees. The authors further acknowledge the small size of their sample and refer to their study as one with a "pilot" character.
von Lersner, U. et al. (2008) ‘Mental health of returnees: refugees in Germany prior to their state-sponsored repatriation’, BMC Int Health Hum Rights, 8: 8–8. https://doi.org/10.1186/1472–698x–8–8.	The explored link is not between specific laws/policies and mental health impacts.	The study explores the experience of individuals who will return to their country of origin after having lived in exile in Germany for a number of years and compares their mental health to the mental health of individuals who have decided to remain in Germany (stayers). The results show that psychological distress among the returnees is high before return, but lower in comparison to the stayers. However, the results are not interpreted in relation to the participation in the voluntary return program.
Walther, L. et al. (2020) ‘Living Conditions and the Mental Health and Well-being of Refugees: Evidence from a Large-Scale German Survey’, Journal of Immigrant and Minority Health, 22/5: 903–13. https://doi.org/10.1007/s10903–019–00968–5.	The authors explicitly state that causal claims cannot be made on the basis of their study.	The study investigates how different living conditions in the context of integration policies relate to psychological distress and life satisfaction among refugees in Germany. The results show that uncertain legal status, separation from family, and living in refugee housing facilities are associated with higher levels of distress and decreased life satisfaction. The authors acknowledge the limitations of the study and that their study design does not allow for conclusions about causal relationships between living conditions and mental health and well-being.
Walther et al. (2021) ‘Mental Health and Integration: A Qualitative Study on the Struggles of Recently Arrived Refugees in Germany’, Frontiers in Public Health, 9: 17–17. https://doi.org/10.3389/fpubh.2021.576481.	The explored link is not between specific laws/policies and mental health impacts.	The study explores the relationship between integration processes and mental health problems among adult refugees in Germany. The thematic analysis presented by the authors highlights a lot of post-migration living stressors that negatively impact mental health. Some of these stressors are related to different policies, but most of them are broader and do not necessarily relate to policies but to integration as a broadly understood phenomenon.
Whitsett, D., Sherman, M. F. (2017) ‘Do resettlement variables predict psychiatric treatment outcomes in a sample of asylum-seeking survivors of torture?’, International Journal of Social Psychiatry, 63/8: 674–85. https://doi.org/10.1177/0020764017727022.	The explored link is not between specific laws/policies and mental health impacts.	The study explores whether resettlement factors predict outcomes of a mental health intervention for asylum-seekers who are survivors of torture. The results suggest that housing conditions have a moderate influence on treatment and patient with access to stable and uncrowded housing show greater reductions in depression, anxiety, and PTSD symptoms over the course of treatment.
Wilson, B. K. et al. (2023) ‘"Letting die" by design: Asylum seekers' lived experience of postcolonial necropolitics’, Soc Sci Med, 320: 115,714. https://doi.org/10.1016/j.socscimed.2023.115714.	The explored link is not between specific laws/policies and mental health impacts.	The study describes the experiences of three asylum seekers to demonstrate the impact of US policies and politics on their physical and mental health. The authors use life stories to present pre-, peri‑, and post-migration stressors. The evidence on mental health impacts is relatively limited, does not appear universally in all three cases, and the author does not link the symptoms necessarily to US immigration policy, but to a complexity of factors (e.g. pre-migration trauma).
Wood, M. et al. (2022) ‘Trauma and resettlement: lessons learned from a mental health screening and treatment programme for Syrian refugees in the UK’, International Review of Psychiatry, 34/6: 588–95. https://doi.org/10.1080/09540261.2022.2072191.	The authors acknowledge limitations and do not present their results in terms of a causal link between law/policy and ASRs' mental health.	The study draws on experiences in delivering a mental health screening and treatment program for resettled refugees in the UK to make suggestions for future policy development. It does not elaborate on any causes of mental health problems related to the UK resettlement policy.
Ziersch, A. et al. (2017) ‘Exploring the Relationship between Housing and Health for Refugees and Asylum Seekers in South Australia: A Qualitative Study’, International Journal of Environmental Research and Public Health, 14/9: 20–20. https://doi.org/10.3390/ijerph14091036.	The explored link is not between specific laws/policies and mental health impacts.	The study evidences mental health issues among asylum seekers and refugees in Australia. The authors identify multiple sources of these issues, some of them related to the housing situation of asylum seekers and refugees. The problems that individuals face in this respect are affordability, not having referees, bad quality housing, insecurity of tenure, etc. Overall, the article focuses on the relationship between housing and (mental) health, and not on the link between specific law(s) or policy/ies and mental health.
Ziersch, A. et al. (2021) ‘Temporary Refugee and Migration Visas in Australia: An Occupational Health and Safety Hazard’, International Journal of Health Services, 51/4: 531–44. https://doi.org/10.1177/0020731420980688.	The explored link is not between specific laws/policies and mental health impacts.	The study explores the experiences of workforce exploitation and its impact on the health of people living on temporary visas in Australia. The authors argue that exploitation, discrimination, underpayment, overwork, and hazardous workplaces have negative health effects on individuals, particularly in relation to mental health.

## Appendix A Search matrix

Twenty-eight keywords were used to search Web of Science, Scopus, PsycINFO, and PubMed. Truncation was used to broaden results. Keywords were combined using Boolean operators. Three search permutations were employed:

### Search 1

“asylum seeker*” OR refugee* OR asylee* OR “forced migrant*”

### Search 2

law* OR polic* OR legislation* OR “immigration law*” OR “immigration polic*” OR “migration law*” OR “migration polic*” OR return OR “durable solution*” OR integration OR resettlement

### Search 3

“mental health” OR “mental illness” OR PTSD OR “Post*traumatic*stress*disorder” OR anxiety OR depression OR “well*being” OR “psychiatric disorder*” OR “psychiatric disease*” OR “psychological symptom*” OR stress OR “mental disorder*” OR “depressive disorder*”

For NexisUni and Legal Intelligence, the search syntax had to be adapted because these platforms do not support complex Boolean strings or truncation in the same way as bibliographic databases. Therefore, the three keyword clusters were entered separately, and results were manually combined and screened.

## Footnotes

1 This timeframe reflects the prominent increase in legislative and policy initiatives of Western governments in relation to the arrival and presence of ASRs (De Haas, 2023, pp. 6-8). The year 1992 was chosen because it marks the beginning of the Bosnian war which eventually led to a large displacement in Europe.

2 We use the term “Western countries” – acknowledging its limitations – to refer to the following countries with a shared history of European colonization and cultural influence: the Member States of the European Union, the United Kingdom (UK), Norway, Iceland, Switzerland, the United States (US), Canada, Australia and New Zealand.

3 Note that as “year of publication” we consider the year of *initial* publication, which might be different than the year indicated in the reference of the respective study. This is often the case for articles included in a special issue at a later stage, such as Fleay C. & Hartley L. (2016), Kahn, J. and Alessi, E.J. (2018), Caroppo et al (2021), Llewellyn (2020), Minero et al (2022), and Chaffelson et al (2023).
